# Understanding the needs and frustrations in adolescent idiopathic scoliosis: a qualitative interview study

**DOI:** 10.1186/s12891-026-10210-7

**Published:** 2026-08-01

**Authors:** Kamila Sykorova, Nenad Pavel, Anna Mathew, Marianne Bakke Johnsen, June Ullevoldsæter Lystad, Helen Christine Bull, Parisa Gazerani, Mette Fløystad Kvammen, He Hong-Gu, Julia Jacoby, Minna Annika Pikkarainen

**Affiliations:** 1 Department of Rehabilitation Science and Health Technology, Faculty of Health Sciences, Oslo Metropolitan University (OsloMet), Oslo, Norway; 2 Department of Product Design, Faculty of Technology, Art and Design, Oslo Metropolitan University (OsloMet), Oslo, Norway; 3 Department of Psychology, University of Oslo, Oslo, Norway; 4 Division of Mental Health and Addiction, University of Oslo, Oslo, Norway; 5 Department of Life Sciences and Health, Faculty of Health Sciences, Oslomet University, Oslo, Norway; 6 Department of Sociology and Social Network, University of Agder, Kristiansand, Norway; 7 Norwegian Spine and Back Pain Association, Oslo, Norway; 8 Alice Lee Centre for Nursing Studies, Yong Loo Lin School of Medicine, National University of Singapore, Singapore, Singapore

**Keywords:** Adolescent idiopathic scoliosis, Health care service, Needs, Frustrations, Difficulties

## Abstract

**Background:**

Scoliosis is a common term used for a group of conditions that involve abnormal curvature and rotation of the spine, thorax, and trunk. If untreated, scoliosis may lead to severe trunk deformities, which consequently limit physical function, exercise capacity, general well-being, ability to work, and the mental health of affected individuals. Little is known about how adolescents with Adolescent Idiopathic Scoliosis (AIS) and their caregivers cope with scoliosis and what kind of needs, frustrations, and difficulties they experience during treatment.

**Aim:**

This study aimed to explore the difficulties, needs, and frustrations experienced by adolescents with AIS and their caregivers.

**Methods:**

An exploratory qualitative design was adopted. A total of 17 participants were recruited through purposive sampling with the aid of user organizations and hospitals in Norway from April to August in 2023. A total of five audio-recorded, semi-structured focus group interviews were conducted, transcribed verbatim, and analyzed using thematic analysis. The discussions focused on the needs, frustrations, and challenges of adolescents with AIS and their caregivers.

**Results:**

This study explored the needs for improvement in health care services related to AIS in Norway, from the perspectives of adolescents and caregivers. Participants reported a lack of communication and information surrounding AIS diagnosis, forcing them to actively seek help. Gaps were also reported, in post-operative follow-up care and conservative treatment of scoliosis, where adolescents with AIS feel overlooked and alone in their recovery and physiotherapy. Participants voiced frustrations, including the psychosocial impact of the condition which affects school attendance due to back pain and absence from activities, leading to feelings of isolation.

**Conclusion:**

The study identified significant gaps in Norway's AIS health care services that could be addressed with digital solutions. Adolescents with AIS and caregivers reported inadequate communication, poor post-operative care, and struggles with conservative treatment.

**Supplementary Information:**

The online version contains supplementary material available at https://doi.org/10.1186/s12891-026-10210-7.

## Introduction

Scoliosis is defined as a structural three-dimensional spinal deformity and is among the most common musculoskeletal disorders. It affects the natural physiological curvature of the spine and causes painful deformities. AIS is the most frequently diagnosed type of scoliosis. AIS affects 0.5–5.3% of adolescents aged between 10–17, predominantly females, [19]. Conservative treatment plays a crucial role in managing scoliosis, especially for mild (10°–25°) and moderate (25°–45°) curves, as it aims to prevent further progression and reduce the need for surgical intervention. Core components of conservative treatment include active involvement from adolescents and caregivers, education, psychotherapy, ongoing outcome monitoring, and adjustments to treatment methods [2, 10, 24].

Adhering to the treatment is essential, as studies indicate that untreated AIS can lead to severe health issues, including reduced lung capacity, decreased physical activity, back pain, or heart diseases [12, 27]. There are studies reporting AIS as a risk factor for developing psychiatric disorders (e.g., anxiety, depression, neuroticism, behaviour disorders) among young patients and causing social problems. However, few studies have explored how adolescents with AIS navigate these burdens, underscoring the need for qualitative research capturing their lived experiences.

Pain in AIS differs notably between conservative and surgical treatment. Post-operative pain following surgical correction tends to be more acute and intense due to surgical trauma and recovery processes. While this pain is typically transient, it requires active medical management and can affect mobility, emotional stability, and reintegration into school or social activities. Although surgery often results in long-term symptom relief, the initial post-operative phase introduces its own psychosocial challenges [3, 30]. This form of pain, although less severe in physical intensity, can significantly impact adolescents’ self-esteem, peer relationships, and daily functioning.

Additionally, adolescents with AIS face much significant physical and psychological stress, which could be alleviated by activating social support systems that help them cope with their challenges Li et al., [14]. Given the emotional and informational challenges associated with AIS, support groups and online forums represent an important complement to clinical care by providing encouragement and peer-based guidance [10, 21]. In a recent systematic review, Hannink et al. [12] summarize the experiences of adolescents with AIS in their daily life situations. Their findings point out that there is a need for clear communication and shared decision-making for treatment and monitoring to manage changes in body shape and activities. These findings highlight broader communication and support gaps in AIS community that extend beyond treatment itself and begin at the point of diagnosis.

In a study conducted by Wellburn et al. [34], parents of adolescents with AIS shared their frustration over not receiving a proper explanation of the disease and that all therapeutic options were not described to them. Participants expressed an overall desire for better provision of written information, tailored individually to the adolescents. When receiving a scoliosis diagnosis, it is often the first time adolescents and caregivers become aware of this condition. They may have many questions that are not possible to discuss further in a short doctor's appointment. In research by Sapountzi-Krepie et al. [28] only 5% of patients said they had time to talk with a doctor about their problems, while 90% stated they wish to have this opportunity. Additionally, their caregivers (mostly parents) tend to feel lost, anxious, and worried due to a lack of information [33]. Wang et al. [33] are advising to pay attention to caregivers’ mental health when diagnosing and treating adolescents with AIS. These findings indicate persistent gaps in communication, information provision, and psychosocial support across all stages of AIS care, underscoring the need to better understand how adolescents, caregivers, and health care personnel experience these challenges—and how technology might help address them.

Despite growing evidence on the physical and psychosocial burden of AIS and the importance of communication and support in treatment, there remains a lack of research describing the specific difficulties, unmet needs, and frustrations experienced by adolescents with AIS, their caregivers, and health care personnel—particularly in relation to how technological tools could address these gaps.

This study aims to describe the difficulties, needs, and frustrations experienced by key stakeholders in AIS care – adolescents, their caregivers, and health care personnel in Norway. Special emphasis is placed on identifying areas where technological support, such as mobile applications (apps), could provide solutions, informed by reflections and insights from IT experts.

## The study

### Design and sampling strategy

The qualitative study described in this paper was conducted in Norway. A total of 17 participants (one medical doctor, two physiotherapists, eight adolescents with AIS, four caregivers, and two information technology (IT) experts were included in five focus group interviews that allowed for both physical and hybrid participation. Participants’ characteristics are presented in Appendix 1. Participants attended the focus groups according to their availability and ability. The findings are part of a broader research effort, with additional results published elsewhere [29]. Focus group interviews were employed, as this method was expected to facilitate in-depth discussions, produce a variety of viewpoints, and motivate participants to share more extensively than in individual interviews. Data collection was continued until thematic saturation was considered to have been reached, at which point recurring patterns and insights were consistently observed across the groups, indicating that the inclusion of further data was unlikely to yield additional conceptual contributions. The diverse, multidisciplinary composition enabled rich, dynamic interactions and the expression of individual perspectives [18]. This method was particularly effective when introducing the app prototype, as the discussions were engaging, developed over time, and yielded valuable feedback.

Those who did not live in Oslo had the possibility to participate in the discussion online. We acknowledge the importance of ensuring participants’ psychological well-being, particularly in a hybrid setting. To address this, consent forms were sent to the participants prior to the workshops. The participants were informed about the nature of the study and had the opportunity to ask questions before consenting. Workshops were led by well experienced researchers who also asked questions from the participants, decided about the speaking order, and showed design prototypes of the potential solution (Appendix 3 and Appendix 4) making sure that all participants had the possibility to contribute and talk about their experiences, perceptions, and to ask questions. In the workshops, participants were reminded about data management procedures, including the secure storage and anonymous use of data, and were assured that their participation was voluntary and confidential. A comfortable and supportive environment was encouraged during the workshops, with participants free to take breaks or discontinue at any time. Contact information was provided should any questions be raised after participation. Data collection continued until thematic saturation was reached, that is, when no new themes or insights emerged from the focus group discussions. The first version of the app prototype was introduced during the fourth focus group, and a revised version, incorporating prior feedback, was presented in the fifth. At that stage, participant responses became repetitive, reinforcing earlier findings rather than contributing new themes, thus indicating saturation. Appendix 1 provides an overview of the composition and structure of the focus group participants.

Participants were recruited through various strategies using a purposive sampling approach, aiming to include individuals who could provide rich and relevant insights into lived experiences related to AIS. Stakeholders from different backgrounds participated to capture a broad range of perspectives. The research team directly invited IT experts and health care workers to participate, to ensure representation of professional perspectives that extend beyond those of adolescents and caregivers. Adolescents enrolment was supported through a partnership with “Ryggforeningen i Norge”, The Norwegian Spine and Back Pain Association, which utilized its broad network for outreach. “Ryggforeningen i Norge” served as an appropriate recruitment source due to its extensive network of adolescents diagnosed with AIS and their families. By recruiting through Ryggforeningen i Norge, we were able to reach adolescents with AIS from varied cultural and socioeconomic backgrounds, as the organization maintains a broad and diverse membership network representing families from across Norway. Diagnosis of AIS was the inclusion criteria for participation in this study. This sampling strategy was appropriate for a qualitative focus group design, as it allowed for the inclusion of participants who could meaningfully contribute to understanding experiences and needs related to AIS.

Information about the study was disseminated via the organization’s website and at multiple events, with interested individuals encouraged to reach out to the research team. This is a user organization dedicated to assisting individuals in receiving proper treatment and advocating for influence over authorities and the health care system in Norway. In total, five adolescents with AIS, accompanied by a caregiver, and three experienced adult user representatives affiliated with the organization took part in the study. The adolescents with AIS cohort included individuals from a range of cultural and socioeconomic backgrounds. Eligible participants represented adolescents with the diagnosis of AIS who had received either conservative treatment or undergone surgery, one participant was under observation without active intervention.

### Data collection

The focus group interviews were conducted in English and Norwegian between April and June 2023. Each session lasted approximately 3 h with a short break in the middle.

The sessions were moderated by experienced researchers, who posed questions and facilitated the discussion using a semi-structured interview guide (Appendix 2) to ensure that all participants had the opportunity to share their experiences. The moderators employed facilitation techniques and used Miro board to actively engage all group members, including those who were less vocal. Open-ended questions were used to prompt discussion, and sufficient time was given for participants to respond thoughtfully. A digital app prototype was presented during the fourth and fifth workshop, which shaped part of the discussion and informed participant responses related to digital solutions (Appendix 3 and Appendix 4). When necessary, the moderators gently encouraged quieter participants to contribute, while maintaining the natural flow and dynamic of the conversation. The speaking order was not predetermined; participants were encouraged to speak freely and respond organically. The moderators ensured that all participants had the opportunity to contribute by gently guiding the conversation when necessary.

All workshops were audio recorded with participants’ permission. Adolescents under 18 years of age participated in the sessions with their caregivers. All the participants signed a written consent form. An English version of the interview guide with semi-structured questions was developed by the research group, validated with scoliosis medical experts from Norwegian hospitals, and is available as a supplementary file (Appendix 2).

### Ethical consideration

The study was approved by Sikt, the Norwegian Agency for Shared Services in Education and Research, with reference number 743837. This study was conducted in accordance with the principles of the Declaration of Helsinki, and written informed consent was obtained from all participants. Ethical approval was not required for this study (584004) according to the decision of the regional committees for medical and health research ethics in Norway (REK).

### Data analysis

All collected data was transcribed verbatim into English. The first author performed thematic data analysis [5] to identify common themes. The aim was to provide a coherent interpretation of patterns and themes grounded in the dataset while maintaining an active researcher’s role. The development of the themes evolves throughout the six-phase analytical process applied flexibly to fit the data. First, researcher 1 familiarized herself with the data by reading and re-reading the transcripts to gain an in-depth understanding of the content, to note initial observations. Second, using an inductive approach, initial codes were generated in NVivo to capture meaningful segments of the data. Third, these codes were organized into potential themes and subthemes using Microsoft Excel Office 365 to facilitate comparison and clustering. Fourth, themes were reviewed and refined through iterative discussions with two other researchers and visual mapping in Miro and visual mapping in Miro to ensure coherence and relevance to the aim of the study. Fifth, each theme and subtheme was clearly defined and named to reflect its essence and relationship to the dataset. Finally, a narrative account was produced, supported by illustrative quotes, to present the findings in relation to the research objectives. The analysis was conducted iteratively, which led researchers to move back and forth through the phases and brought new interpretations of the data. Some of the codes appeared to have been used multiple times. Consequently, to strengthen trustworthiness, two independent researchers (researchers 2 and 3) reviewed the analysis and shared their feedback. The datasets generated and analyzed during the current study are not publicly available due to the confidentiality of the study but are available from the corresponding author on reasonable request.

### Rigour

Methodological rigour was upheld through systematic documentation of analytic decisions, ongoing reflexive engagement throughout the research process, and adherence to established qualitative standards to ensure credibility, dependability, and transparency of the findings. Trustworthiness was further supported by following the criteria proposed by Lincoln and Guba [15]. By providing a comfortable environment during focus group interviews, the truth value of collected data was enhanced. Participants were informed beforehand that their personal data would be kept confidential. Data consistency was assured during the collection process by audio-recording the workshops, transcribing the verbatim, and keeping the records. The whole process was conducted by researchers keeping a neutral stance. The reliability of the findings was strengthened by analyzing and generalizing the collected data. In addition, the initial data analysis process was reviewed by two other co-authors independently.

## Results

The study involved five focus group sessions with a total of 17 participants representing a diverse range of perspectives (shown in Appendix 1). The groups were intentionally composed to include:

- Adolescents diagnosed with AIS (*n* = 8)
- Caregivers (*n* = 4)
- Health care personnel involved in scoliosis management, such as physiotherapists, orthopedic specialists, or nurses (*n* = 3)
- IT experts (*n* = 2), invited as stakeholders in the digital co-design process

The interdisciplinary structure allowed for a broad exploration of the challenges and opportunities associated with AIS treatment from both lived experience and professional viewpoints. While adolescents and caregivers provided insights into daily life with AIS and its psychosocial impact, health care personnel contributed to clinical context and reflections on evolving treatment practices. IT experts, included in later workshops, offered perspectives on the feasibility of technological interventions aligned with adolescents needs. Their contributions were limited to discussions on future digital solutions and are reported more fully in a separate publication [29].

In this paper, data from adolescents, caregivers, and clinicians form the basis of thematic analysis, while feedback from IT experts is referenced only where it directly relates to user experience or expectations in the AIS care process.

**Fig. 1:**
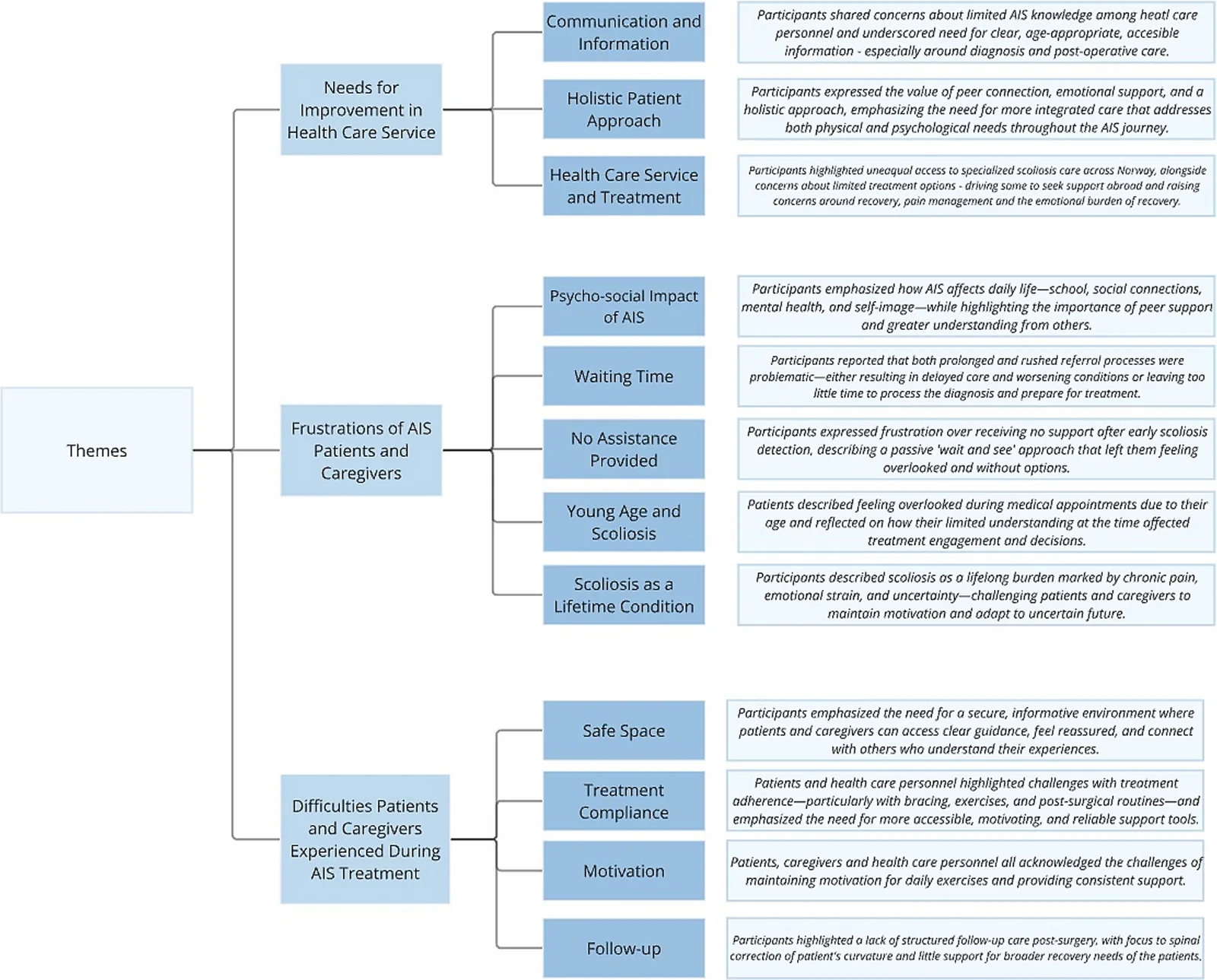
Themes and subthemes identified through thematic analysis of five focus group interviews with explanatory descriptions for each subtheme

Based on the thematic analysis of collected data, three themes were generated (see Fig. 1): (1) Needs for Improvement in Health Care Service, (2) Frustrations of AIS Adolescents and Caregivers, and (3) Difficulties Adolescents and Caregivers Experienced During AIS Treatment. Due to the high saturation of data, subthemes were developed to provide detailed information on the individual themes.

### Theme 1: needs for improvement in health care service

This theme summarizes both adolescents’ and their caregivers’ needs in health care services provided in connection with AIS in Norway.

#### Subtheme 1.1: communication and information

According to adolescents’ and caregivers’ experiences, there is a lack of communication and information provided when receiving an AIS diagnosis. Some of the adolescents described that their general practitioner, physiotherapist, or health care personnel at the local hospital had no knowledge regarding their AIS condition.

Adolescent: But when I was at my own doctor, fastlege (translated as GP General practitioner)… He did not really know anything about scoliosis. And he had never seen anything like that.

Adolescent: And it's the same with the doctors. I feel like doctors at my local hospital don't really know about scoliosis. You have to talk to a specialist.

In response to these concerns, a surgeon participating in our data collection acknowledged that awareness of AIS among health care personnel has improved over time. They are actively working to disseminate knowledge about AIS among health care personnel.

Scoliosis expert: And tomorrow we have a special day for physiotherapists from all around the country to…That is called a fagdag (translated as professional development day) for scoliosis in physiotherapy. So we try to spread the knowledge as good as possible.

Participants felt a great deficit in the quality and amount of information provided, and had to actively seek help from themselves.

Researcher: How did you experience switching to a new regimen of training? Could you tell us more about it? Adolescent: I really feel like I had to find most of it out on my own.

Scoliosis Expert: Then they start to look… look for the information on the Internet… Researcher: Are they? Scoliosis expert: They… they told me. Really. The the worst things comes first. So the… the worst scenarios pop up on the internet and… It's not relevant information and they need some way to to access this. By some trusted sources and somebody they can trust and… and that… that… that is reliable information.

It seems also to be crucial to provide proper information at the right time. For example, being referred for surgery often causes shock for adolescents and their caregivers, so they are not able to focus on absorbing the information given.

Caregiver: We couldn't take that at once. We had to say that this was not possible. We had to ask for a new time. We had to go home. To be able to process it. It was completely unpredictable.

According to participants’ experience, another challenging situation is when they are given further instructions straight after the surgery. It is difficult to remember anything due to the strong medication.

Scoliosis expert: After surgery… You are so confused of everything that they probably, no matter what information they get, they don't remember afterwards. So that's one one big challenge there..

Scoliosis expert: So, it's… it's just so confusing situation. A lot of medication, a lot of things are happening, and they're worried and all that. So, it's a it's it's just the situation itself.

Scoliosis expert: And also, if there's a surgery and after surgery, yeah, they… they probably get a lot of information, but they can have a strong medication and cannot remember anything afterwards. So maybe giving them this… this exercises and information.

Although one adolescent also highlighted positive experiences:

Adolescent: Yes, I feel like I have a very good relationship with my surgeon. I talked to him only a few months ago and it's over three years since I've had the surgery. So, it's very open and I can always call if I'm wondering about something.

#### Subtheme 1.2: holistic approach of adolescents with AIS

The absence of emotional support throughout the scoliosis’ journey emerged as an important theme. All adolescents wished for community support, understanding, and sharing experiences with others with AIS. Caregivers and scoliosis experts shared this perspective, emphasizing the importance of peer connection and shared knowledge in improving adolescents well-being. Nowadays, this is typically facilitated through group physiotherapy sessions offered to some AIS adolescents, providing them with the opportunity to interact with others.

Caregiver: But I think it is more important to talk to experienced people who understand what you are going through and who can give advice.

Scoliosis expert: Just think about the scoliosis brace patients they meet, they want to connect, they want to share information. They want to get answers to the questions, and we can be sure and safe that they will get this information in a good way.Exper

Scoliosis expert: From a physiotherapist who is specialized in scoliosis. That’s just about meet and understanding, like X (adolescent’s name). It gave a lot. It is psychology. To talk to someone who has experienced the same.. Is better than going to a psychologist.

Participants repeatedly stated the belief that having the opportunity to speak to someone who had undergone a similar treatment journey would probably have helped them in making decisions regarding their treatment choices.

Adolescent: When it comes to communication it should support the possibility to chat with people or young people in a same situation like you mentioned.

According to adolescents’ and caregivers’ reports, the health care services never provided them with any psychological help.

Scoliosis expert: If that was to happen now, we would have done that in a different way. Because we now have a really good collaboration with the psychologist.

Adolescent: Because I'm very glad that you are having different specialties like psychologist and… Because that was never a theme when I was young. So, and I think that's important that also the physiotherapists work together and not just the surgeon.

During doctor appointments, discussions typically centered on the curvature of the spine and back-related issues. Most of the adolescents, however, shared a desire to be more holistically approached.

Adolescent: I do miss a bit that they don't see you as a whole person with like a mental health as well. You just see an x-ray and you need a back... and you have a back you have to fix.

Adolescent: But you don't really get anyone to talk to once you're going through it. It's like very much focused on your physical health instead of both.

One scoliosis expert acknowledged the need for a multidisciplinary approach, including the integration of a psychologist into care services. However, collaboration with an occupational therapist was not currently in place.

Scoliosis expert: We have used the psychologist here to educate ourselves in order to meet the different and difficult situations.

Scoliosis expert: And we have also collaboration with our child psychologists. And they have … they have tried to train us. They have been with us at our outpatient clinics.

#### Subtheme 1.3: health care service and treatment

Not only adolescents and caregivers, but also health care personnel acknowledge that the provision of health care services is not equal for all adolescents across Norway. There are three hospitals in Norway treating children and youth with scoliosis (Rikshospitalet in Oslo, St.Olavs Hospital in Trondheim, and Haukeland hospital in Bergen). Many of the interviewed adolescents had to travel to Oslo to receive standardized scoliosis treatment as their local hospitals were unfamiliar with AIS treatment and didn’t have enough experience.

Scoliosis expert: It's not that easy to get the same treatment when you live in the north of Norway.

Adolescent: Yeah, I went to a local physical therapist, but I don't really feel like they had a lot of knowledge about scoliosis. I've been to very many local physical therapists and almost no one had a lot of knowledge. So, the best physical therapist that I've been to was at Oslo University Hospital.

Some of the respondents were not satisfied with the health care offered in Norway and sought care abroad.

Caregiver: But in Norway they didn't offer anything else than surgery.

Adolescent: I'm from a really small place, so we just have like almost one doctor and one physiotherapist. She was reading a lot of information about scoliosis, but I didn't do any specific exercises since I was doing what I got from X (name of the city outside of Norway). So I would just get a massage and that was pretty awesome actually… But yeah, I think that I have a big bad experience with that one, to be honest. So actually this kind of scoliosis-specific guidance, help would be really needed somehow.

Adolescent: So, in the end of May 2013 me and my mum went to X (name of the clinic) Scoliosis Clinic in X (name of the city outside of Norway).

Adolescent: I would never be without the strict training beforehand. Or I personally think that my case would have turned out different. If I didn't went to X (name of the city outside of Norway)… Or if my mom didn't check for a physiotherapist. Who actually know what scoliosis was.

According to the medical expert this creates a problem, as when these adolescents return later with heavier curvature, bigger surgery is required than before.

Scoliosis expert: We do not like have parallel follow-ups here. We said that of course if you are Norwegian citizen, you are welcome to be seen at our hospital when you are finished with your… With your treatment abroad. So then.. and then we take them in and we… do the surgery.

Undergoing surgery on adolescents’ backs is a major, emotionally taxing decision to make, connected with anxiety and feelings of fear. Therefore, some of the participants refused this way of treatment suggested to them, which led to escalation of their spine curves and worsening of their case.

Adolescent: I was only 14, so I don't think I really understood. But I remember being scared. And also, when I was going into my second surgery, I was very scared because of what happened last time.

Adolescent: But we wanted to try everything else first. Because if I got operated when I was 12, I might have done the surgery several times until I was outgrown.

Adolescents described a lack of adequate follow-up, especially in the post-operative phase. Undergoing surgery means the curvature of the spine is fixed; however, adolescents need a longer period to recover. One adolescent still experienced pain many years after surgery, and another described discomfort and needed a longer time to heal than what was expected by hospitals.

Caregiver: From a medical report a year after the operation you are recovered, but at first you weren't healed, and there was a lot of pain.

Adolescent: I also think it's very stupid to have the impression that when you're operated on, you're fixed.

Adolescents and caregivers expressed a desire to receive help with pain management and be informed regarding their rights on where to get help and aid afterward.

Caregiver: They did not set up an ergo therapist, or any other aids at the school. She was not allowed to go to school. She got worse days, and worse grades. We had nothing. Nothing at all.

Caregiver: The support or the offers are taken away, that you can get some assistant help for three months, you can get a double set of books, you can get school taxis, and things like that.

Health care personnel also acknowledged these concerns, emphasizing the importance of providing clear guidance and support to ensure adolescents receive the necessary care.

Scoliosis expert: But when they are new to surgery or have a lot of pain and can't get to this… the biggest need is others. Experienced people. Whether it is adults or scoliosis patients. Someone who can give advice and tips.

### Theme 2: frustrations of adolescents with AIS and caregivers

This theme summarizes both adolescents’ and caregivers’ frustrations of coping with scoliosis in everyday life.

#### Subtheme 2.1: psycho-social impact of AIS

Scoliosis also affects school attendance. Due to back pain, adolescents need to take shorter days at school or experience big absences due to the long post-operative recovery period, social isolation, and academic challenges. Our participants shared their wishes to receive greater understanding and support from teachers.

Adolescent: I actually lost a lot of school. I had to take my high school six months overtime. I couldn't finish all my classes. So, I had to take them on my own.

One adolescent faced a challenge of being understood by her peers due to the brace treatment as her appearance was affected.

Adolescent: Yeah, but I agree with X (adolescent’s name) about being open. So, we… so we had a presentation for the class and for the whole school to tell about the situation. So, I think that's a good thing to do if you… Yeah. Because then they will understand.

Another important subject that emerged from scoliosis workshops was pain management which can last for a long time. Pain is individual and can occur within conservative treatment but also in the pre-operative or post-operative phase. Some adolescents mentioned struggling to find a comfortable sitting or lying position to sleep.

Adolescent: I feel like I have to find a way to live with it. I think I have a bit more pain than what's normal.

Adolescent:..and there, someone is completely fine, and I've also talked to someone who's like me and still are in a lot of pain, even though it's a long time since the surgery, and you should probably have recovered by now, but I feel like I have to find a way to just live with the pain.

IT Expert: It.. it causes a lot of mental problems when you hurt so badly.

Having scoliosis significantly affects the everyday life of the adolescents. Their absence at school or from activities, and the need for daily exercising means not seeing their friends as much as they would like to. By receiving a diagnosis, they feel different compared to their peers and seek understanding. Adolescents acknowledged that it helped to talk to someone else who has been diagnosed with scoliosis, to meet other adolescents at group therapies or participate in the meetings organized by the Norwegian Spine and Back Pain Association “Ryggforeningen i Norge”.

Adolescent: I met one who is almost my age. Just that we could talk about how it is at school. To have that similarity there. That is the best thing a psychologist can do.

#### Subtheme 2.2: waiting time

This subtheme has been mentioned repeatedly regarding both long waiting time and short waiting time. Many adolescents and caregivers found it frustrating to wait a long time to be referred to a specialist or for surgery, as the spine curvature can progress rapidly.

Adolescent: It gets all too bad. I probably wouldn't have to ended up getting surgery two times if I wouldn’t had ended up waiting too long.

Caregiver: We went to Ahus and met orthopedist, and I was told she had scoliosis like 34 degrees, and we were just put on hold. And we did not get so much information about scoliosis what it was. So, I was going home and doing a lot of reading, and we felt like no, we should not wait. And then we contacted by ourselves Rikshospitalet to get some more.

Caregiver: And then we had to do a lot of calling to get her to an appointment. And then she had like X (adolescent’s name) told 55 degrees already in like 6 months' time.

On the other hand, some adolescents had been referred for surgery so quickly after diagnosis that they did not have enough time to cope with their current situation.

Caregiver: We experienced the opposite. We thought it went so fast.

#### Subtheme 2.3: no assistance provided

Some adolescents described their situation as “being put on hold”. Caregivers expressed their frustration at getting no help for their children, although they discovered scoliosis at an early stage and wished to prevent curvature from growing.

Adolescent: I was told to wait and see. But then we realized that it probably would just get worse.

Researcher: So, you've only received… what kind of treatment have you received? Adolescent: In Bergen, there was absolutely nothing.

#### Subtheme 2.4: young age and scoliosis

Adolescents shared their experience of being overlooked at doctors’ appointments due to their young age. According to their experience, health care personnel primarily engage with caregivers in conversation.

Adolescent: Asking questions to them and not me. So, I felt a bit left out even though everything was about me. So, yeah, it was a bit strange actually.

Adolescent: I do not think I quite understand what was going on. Like X (adolescent’s name) said. They were always talking to my parents instead of me.

Some adolescents also acknowledged they were too young to understand the severity of their situation or the importance of treatment compliance.

Adolescent: I was young, and I didn't understand how important it was. And I think that's the main reason actually.

Adolescent: I was, you know, just a child. I remember not really understanding what I was going to go through.

#### Subtheme 2.5: scoliosis as a lifetime condition

Scoliosis represents a lifelong health condition. For adolescents, it means daily exercise. Adolescents and caregivers shared their frustration at keeping motivated to follow this strict regimen for this long.

Adolescent: Now I totally lost the motivation. I was tired of training every day.

Researcher: But it's really years of usage if the condition is just a… kind of lifestyle that you have to live with in a way? Adolescent: Yes.

Doubts arise whether adolescents’ dedication to training hard or wearing the brace 23 h a day will prevent them from undergoing surgery. Adolescents also wonder what their future is going to look like, regarding their work life, pregnancy, etc.

Adolescent: So even though my journey is like kind of over after surgery they say… I still think about my scoliosis every day.

Adolescents suffer from long-lasting pain, trouble breathing, and other discomfort. For both adolescents and caregivers, these often represent lifelong conditions that are heavy to accept.

Adolescent: I feel like I have to find a way to just live with the pain.

Caregiver: Yes. It never goes away. It's like forever. You have to deal with this new situation. It never goes away.

### Theme 3: difficulties adolescents and caregivers experienced during AIS treatment

This theme summarizes the difficulties that adolescents with AIS and caregivers encounter related to scoliosis diagnosis and difficulties that other workshop participants have observed adolescents and their caregivers facing when undergoing AIS treatment in Norway. Some of the experiences represent clear examples that could be resolved by using digital technology.

#### Subtheme 3.1: safe space

All adolescents and caregivers agreed that receiving an AIS diagnosis was a difficult period to bear. They needed to seek information regarding their new life situation. Together with health care personnel, they agreed that there is a need for a place that would help them overcome their feeling of uncertainty and fear and provide quality information and answers to their questions.

Scoliosis Expert: So that when they get home and can sit down and reflect… Over this message they have got at the hospital, they have a safe place to look into. To to get the information they didn't hear when they got this shocking message from the doctor.

Adolescent: But what I think is good about this is that it creates a kind of security that the exercise is safe to do for my back. That you get a confirmation that, let's have a pull-down, which is a very common back exercise. It is very popular among ordinary people. But then you get that confirmation here via this app that it is safe that my back can handle it.

Adolescents wish they could have shared their experience with someone in a similar situation to better understand their new life situation and prepare them for possible scenarios or help them to make the right decisions regarding their future.

Caregiver: But I think it is more important to talk to experienced people who understand what you are going through and who can give advice.

Adolescent: And when I was 15 years old we found Ryggforeningen. Which has like this meetup group once a year. So everyone who has scoliosis from all around the country could come and meet up. And just talk and yeah share some experience. And I found that quite helpful. It was pretty nice to get to know the children my own age. And hear their stories. So yeah.

Researcher: That period of waiting and uncertainty was really while, while not having support was really problematic for them.

Adolescents are expected to adjust to the new regimen and are obligated to exercise daily for the rest of their lives. It is difficult to collect proper instructions as to what is safe regarding their back condition. They hope for a platform that provides correct recommendations.

Researcher: Why is it difficult to do it at home? Caregiver: It's possible for a while. But it's difficult to do it consistently. Every other day. For a long time

#### Subtheme 3.2: treatment compliance

Another observed finding was the difficulties adolescents face in following their prescribed treatment plans. Those adolescents who are referred to a conservative bracing shared their struggles with their treatment plan.

Researcher: I think X (adolescent’s name) pointed out already that she ignored the brace as much as possible. Maybe we can start from that. Adolescent: Yes. Researcher: And was hot and uncomfortable, I think you said.

Adolescent (talking about the brace): I was supposed to. But I didn't use it at all. I used it when I was going to school and stuff. But I didn't like it.

Physiotherapists stated obstacles when recommending conservative exercise treatment to the adolescents as it is, among others, difficult to recall the movements afterward. As explained by an adolescent, that she kept track of the exercises by writing them down:

Scoliosis expert: Our experience is that if you give people this exercise package and it's up to the parents or to the child or the youth themselves to follow it up, yeah. Then you have to be clever. You have to do that in a very motivating manner to make it happen.

Adolescent: Of course, this is individual, but for me it is strength training that works well, and cardio training. I mean not running, but cycling, ellipse machines, rowing, and things where you get bit more support and are stable. I also wrote down massages, which I got from a physiotherapist. Low stress levels, and breathing exercises, which help to avoid knotting the muscles in the neck due to stress. And breathing exercises, because I have a lot of problems with heavy breath. I know that it is quite common when you have scoliosis.

For adolescents undergoing surgical treatment, it is difficult to remember the instructions they were given in the hospital. The same experience was also confirmed by physiotherapists. Therefore, they would all appreciate a reliable platform that they can refer to at any time, where the post-operative instructions could be stored, or where the adolescents could reach out to health care personnel for help very quickly.

Scoliosis expert: And then they go home, and they feel like I, I don't remember anything what was said. What should I do? How can I get better? And everything is miserable.

#### Subtheme 3.3: motivation

This topic was also well recognized by both health care providers and recipients. Adolescents struggle to find motivation for daily exercising, which health care personnel confirmed by finding it difficult to provide support that keeps adolescents motivated.

Researcher: So, it's understandable that this takes a lot of motivation? Scoliosis expert: Yes.

Researcher: Yeah, but what would you advise others?

Adolescent: I would probably say that it takes a lot of time and I had to be patient, but in the end it will be better than what it was.

#### Subtheme 3.4: follow-up

According to participants’ narratives, a follow-up of adolescents with AIS is very poorly addressed by health care providers in Norway.

Caregiver: Otherwise, there's nothing. But just a follow-up once a year.

In the post-operative phase, the focus is put mainly on the adolescent’s back. If the curvature of the spine is fixed, the adolescent doesn’t seem to require additional follow-up. On the contrary, adolescents expressed frustration over the lack of continued care, as different issues beyond their back arise when recovering from surgery. In these cases, health care personnel depend on adolescents to initiate contact themselves.

Adolescent: I was healed, but after a very short time I got sick leave. They saw that I still had a lot of pain, so they took me in again. And I'm still not healed at all.

Scoliosis expert: But the standard patient with a good result, we don't see them more than after six months.

Scoliosis expert: So, we need to… to… to rely on the patients to take contact if they have…if they have problems.

## Discussion

This is one of the first studies reporting the needs, difficulties, and frustrations that adolescents with AIS and their caregivers face during their treatment journey. The study highlights the need for significant improvements in AIS health care services in Norway, as reported by adolescents and caregivers and other stakeholders (including medical experts, physiotherapists, and IT experts). Issues include inadequate communication and information around AIS diagnosis, poor post-operative follow-up care, and struggles with conservative treatment. The psychosocial impact of AIS is profound, affecting school attendance, causing back pain, and leading to feelings of isolation. Adolescents also struggle with maintaining motivation for daily exercises, a necessary part of AIS treatment.

### Conservative treatment challenges

Our data analysis shows that both adolescents with AIS and their caregivers often need but lack psychological support. According to Wang et al. [33] adolescents with bracing suffer most from psychological burden (including self-image, stress and mental health, and vitality). This has been corroborated by research conducted by Kaelin [13], which highlighted the complications associated with brace treatment mainly revolved around psychological aspects, skin irritation, and a temporary decrease in vital capacity. However, these are outweighed by the benefits associated with brace treatment. A great amount of frustration was uncovered in our research. AIS was described as a complex condition with a significant psychosocial impact on their lives. Adolescents reported that AIS decreased their school attendance and caused more frequent absences from sports and other free time activities, which led to social detachment.

AIS has instant effect on social interactions and relationships. Adolescents in our research provided details regarding this topic, such as having shorter days at school, prioritizing daily exercising over spending time with their friends. One adolescent faced the challenge of being understood by her classmates when she underwent brace treatment. Other adolescents must drop out of their usual activities and hobbies, in some cases also school attendance [12]. Their body self-consciousness is affected, leading them to change their clothing to conceal the spinal curve or brace and to feel ashamed of their postoperative scar [6]. They don’t want to be seen undressed. They want to look and feel normal again and get back to normal life.

Beyond immediate social concerns, adolescents in the study also reflected on the long-term implications of AIS. One participant, for example, expressed concerns about potential challenges related to pregnancy as an adolescent with AIS. A scoping review by Theroux et al. [31] revealed that women with scoliosis have similar pregnancy rates and durations compared to healthy controls and the national average. However, they appear to require assisted reproductive technology more frequently. Back pain was reported in adolescents with AIS during pregnancy, with a higher prevalence in those with lumbar curves compared to thoracic curves. Pregnancy is not considered a prognostic factor for curve progression, but some adolescents with AIS may experience significant curve progression after pregnancy, regardless of the treatment they have received [31]. Additionally, Grabala et al. [11] reported significantly higher caesarean section rates among adolescents with AIS who underwent surgeries (64%) compared to healthy controls (33%), highlighting the potential impact of scoliosis on delivery outcomes.

According to the literature [8, 13], most experts agree that physiotherapy alone has no effect on the progression of scoliosis. Scoliosis-specific exercises (SSE) may be beneficial may be beneficial for adolescents with AIS but there is low-quality evidence. However, in combination with brace treatment, it has been shown as beneficial. According to Marchese [16] patients’ preferences and values play a crucial role in conservative treatment success. By considering these factors, treatments can be 2–3 times more effective compared to the treatment that did not take preferences into account. Marchese’s study highlighted findings from a recent systematic review, which concluded that exercise therapies may provide benefits for both the physiological and psychological aspects of AIS treatment. Marchese et al. [17] described how Schroth therapists worldwide incorporate various adjunctive methods in the treatment of AIS. The approach to Schroth therapy is shaped by the adolescents, their caregivers, the therapist, and the health care systems in which the treatment is delivered, ensuring a person- and family-centered model of care.

If an adolescent is diagnosed with a mild curve and doesn’t reach the criteria for bracing, neither physiotherapy is prescribed. Therefore, it may appear frustrating for adolescents and their caregivers, being offered no treatment at all despite the early detection of scoliosis [13]. This initial frustration is accompanied by additional challenges, as adherence to conservative brace treatment presents its own set of difficulties, which are well documented in the current literature. According to Samad-Soltani et al. [27] mobile-based exercise programs at home promote compliance with rehabilitation compared to paper instruction handouts. Physiotherapists who participated in our data collection also reported clinical experience with out-patient adolescents having difficulties in correctly remembering the recommended exercises or instructions given in hospitals shortly after surgery. At that time, adolescents have plenty of other thoughts in their heads and are strongly affected by medicine. All participants agree that they struggle with motivation in long-term rehabilitation, as AIS represents a lifetime condition. Adolescents must follow strict regimens to avoid the worsening of their curvature and to avoid surgery, which is sometimes not possible despite their serious dedication.

### Surgical treatment challenges

Another discussed topic was pain associated with scoliosis, lasting for a long period of time, and affecting adolescents mental health. Participating in normal hobbies or in everyday interactions like being with friends and families, decreases adolescents’ back pain in the post-operative phase according to Rullander et al. [26]. Participants in her study reported symptoms:new-onset symptoms six months after their surgeries. Using strategy and support has been shown to be essential for pain management. More attention should be directed towards addressing these challenges. For instance, the Norwegian health care management for AIS, which now includes psychologists as part of a multidisciplinary team, is a step in the right direction. However, the absence of occupational therapists is notable and warrants consideration for comprehensive care.

Our research uncovered the need to promote equal health care service across the whole Norway for adolescents with AIS. Some of them had to travel to Oslo to see a specialist or undergo treatment abroad, as they were not satisfied with the treatment options offered in Norway. This seems a reasonable decision as adolescents are very young and scared when they are referred for back surgery only, so they searched for other treatment options together with their caregivers. However, some of these adolescents return later to the Norwegian experts with bigger curvatures requiring greater surgery. Caregivers try to choose the least invasive treatment possible for their children. For most, surgery represents an opportunity to lead a normal life again.

Interviewed adolescents in our study also identified a gap in follow-up care, especially in the post-operative phase. The same reports were obtained by Rullander et al. [25] in their study when interviewing adolelscents with scoliosis. A solution to this can be as suggested by Bottino et al. [4], who reviewed their study of mobile health apps available for AIS. They suggested that apps could provide adolescents with AIS access to health care services for periodic monitoring, reducing the need for in-person visits and including information about the scoliosis condition, relevant for the adolescent’s age group (so that the youngest do not feel overlooked). Additionally, apps could facilitate consultations with doctors, allowing adolescents to receive immediate feedback [4]. In our study, adolescents with AIS also advocated for their rights and requested to be informed about possible aids and equipment that could significantly alleviate their daily challenges. Such information can be efficiently provided through quick telecommunication with their doctor.

### Communication barriers in AIS diagnosis and treatment

In our research, it was found that there is a lack of communication and information provided at the time of AIS diagnosis, and a lack of treatment instructions in both the conservative and surgical treatment journey. Diagnosis of scoliosis brings many questions to the adolescents — *What is my future going to look like?* regarding work, pregnancy, and back problems. It impacts life immediately in terms of social connections.

All adolescents in our study interpreted the same experience related to communication as being overlooked by doctors when discussing possible treatment options. Doctors tend to talk to their caregivers instead, which only promotes their feelings of frustration. Few adolescents realized now that they were too young to understand the importance of treatment compliance when they were prescribed bracing, for example. They think they would have benefited from talking to other adolescents at that time, which corresponds with the findings published by Wellburn et al. [34]. This demand is partly covered in group therapies offered to AIS by some Norwegian hospitals.

Adolescents, together with their caregivers, feel that there was not always clear communication with health care personnel, and this had an impact on their decision-making process. In the systematic review by Motyer’s et al. [20], caregivers of AIS patients have little knowledge regarding their children’s condition. It seems convenient to search for information online, but there is a need to provide better quality information regarding caregivers’ concerns related to their children’s treatment [20]. The dissatisfaction related to scoliosis information obtained by caregivers and patients has been confirmed, among other authors, also by Wellburn et al. [34] and Cheung et al. [6].

Paulson et al. [22] in their research pointed out that using a digital tool can serve as an information channel for adolescents, where presented information is accurate and simplified for adolescents’ understanding (avoiding medical jargon/terms) [22]. According to the medical experts in our study, adolescents who are better prepared for treatment experience better outcomes and faster recovery.

It has been mentioned in the current literature [1] that adolescents often seek information, especially using the Instagram platform, where users with AIS share their stories and hope to get feedback from their followers. Thus, adolescents form online communities not only related to their interests and needs but also regarding their health conditions [32]. Most adolescents with AIS and their relatives would appreciate a more advanced level of provided information. They wish no longer to receive informative flyers or read fact sheets posted online. Their expectations have moved to videos [32]. Misinformation still remains frequent online [32].

### Enhancing support and communication in AIS treatment

Participants in our project described the frustration of having to wait long times before seeing a specialist and receiving help, during which their spinal curvature continued to progress rapidly. Conversely, some participants shared experiences when the process between getting the diagnosis and being referred to surgery went so fast that there was not enough time to cope with the situation. There is a need to improve the provision of support for adolescents during the waiting period, such as psychological counseling or informational guidance, as well as to ensure adequate help is available to those who must quickly adapt to their new diagnosis and treatment plan. Some participants were not offered any form of support, either in terms of medical treatment or psychological care. If there is no available treatment for them, they hope to receive some guidance or psychological support. Participants in Wellburn’s et al. [34] study recommended as a resource support communication with others who have been through the same process. It is significant that the provided support is personalized to the adolescent’s specific situation. Adolescents with AIS seem to use online support frequently, an emotional help especially [22]. Adolescents in our research desire to have a support tool to exchange experiences with other adolescents.

### Smart technology to support adolescents with AIS

Our research uncovers difficulties that adolescents, caregivers, and health care personnel experience within AIS treatment. Participants would appreciate any solution that can serve as a safe space for adolescents and caregivers, where they could share their questions and doubts, and ask experienced adolescents with AIS for recommendations regarding useful, safe treatment and life with scoliosis in general (Wellburn et al., 2021). Both adolescents and caregivers from our study believe that hearing someone else’s story about living with scoliosis would normalize their situation and make them feel less different. Health care providers believe that informed adolescents are better prepared and easier to treat. Samad-Soltani et al. [27] in their study pointed out that the usage of smartphone medical apps can improve adolescents’ preparation by providing proper information and coping strategies.

Smart technology can, according to Samad-Soltani et al. [27], enhance self-management in individuals with musculoskeletal conditions and thus increase motivation. Both health care providers and adolescents in our study shared a similar idea of having a platform with the information they could recall later at any time, and that would enhance motivation. Most of the adolescents also experienced a lack of follow-ups in areas where the real obstacles actually occurred. According to their words, treatment focus was put mainly on their back while they faced different challenges [26]. Cho et al. [7] mentioned that technology, e.g., smartphone apps, can help to find a strategy in the period between follow-ups when patients experience struggle. Smartphones are easy, accessible, and flexible tools. Health apps are feasible and convenient solutions to deliver health care services remotely at any time [27].

A digital app prototype was presented to participants during the final workshop, and feedback was gathered. This interaction informed the digital focus of our discussion and implications, reflecting both participants’ interest and the project’s broader goal to explore technology-based solutions. However, the broader clinical and organizational recommendations outlined above also emerged directly from participants’ narratives and should not be overlooked.

Our research reveals a range of difficulties faced by adolescents, caregivers, and health care personnel during AIS treatment. While participants highlighted the value of digital tools, such as a centralized app for information and peer support, they also identified several non-digital areas needing improvement. These include: (1) improved communication at the time of diagnosis and during follow-up; (2) the need for a more consistent, equitable distribution of services across regions in Norway; (3) the importance of expanding multidisciplinary teams to include occupational therapists; and (4) better psychological support integrated throughout treatment. Addressing these issues could significantly improve the adolescent journey regardless of digital interventions. By identifying these needs, our findings indicate the potential areas for improving AIS care in Norway from different stakeholder views.

The clinical implication of this study highlights how stakeholder perspectives can inform future digital solution that meaningfully support adolescents with AIS in Norway. In our study, adolescents, caregivers, and health care personnel consistently highlighted the need for better coordination between different care providers, particularly in addressing both physical and psychological aspects of AIS management. A recurring theme across the results was the need for more accessible psychological support, particularly to help adolescents with AIS to cope with body image concerns, brace related stress, and long-term motivational difficulties. These findings further suggest that future digital solutions should facilitate communication and coordination across multidisciplinary teams, rather than focusing solely on isolated aspects of care. These factors are consistently recognized in recent AIS research as central to effective conservative management and sustained engagement with treatment [24].

Our findings also reflect well‑documented challenges in current clinical practices at AIS. For example, adherence to SSE is often poorly monitored in routine care, which limits health care personnel’s ability to evaluate treatment effectiveness or tailor rehabilitation plans to adolescent’s needs [9]. At the same time, emerging findings suggest that digitally supported, homebased exercise programs may improve treatment outcomes more effectively than conventional approaches, highlighting the potential value of integrating digital tools into AIS treatment system providing multidisciplinary care [36]. These developments reinforce the potential value of integrating digital tools into AIS care pathways, especially within multidisciplinary treatment settings [35].

Importantly, our results also align with broader recommendations in pediatric rehabilitation, where multidisciplinary collaboration and strengthened social support systems are considered essential for delivering high‑quality, family‑centered care [23]. Prioritizing coordinated teams, such as physiotherapists, psychologists, and nurses, alongside structured support for families has been shown to improve treatment adherence, psychosocial well-being, and overall satisfaction with care. Such approaches help families feel more empowered and supported throughout the treatment journey. This includes improved coordination between physiotherapists delivering SSE, psychologists addressing emotional burden, and clinicians monitoring treatment progression.

By combining these established clinical insights with the lived experiences and expectations expressed by adolescents with AIS, caregivers, and health care personnel in this study, our findings highlight the clear need for more structured, multidisciplinary rehabilitation pathways for adolescents with AIS, including closer integration of physiotherapy, psychological support, and post-operative follow-up. Consistent with previous research showing that adolescents experience substantial psychosocial strain during both conservative and surgical treatment, enhanced collaboration between rehabilitation professionals — such as physiotherapists, psychologists, and occupational therapists — may help address challenges related to pain management, motivation, and social participation. Moreover, improving communication practices across the AIS care continuum, as emphasized in earlier studies, can support shared decision-making and ensure that adolescent and caregivers receive timely, age-appropriate, and comprehensible information. Together, these implications suggest that strengthening multidisciplinary coordination and adopting patient-centered support strategies could meaningfully improve rehabilitation outcomes and overall care experiences for adolescents with AIS.

Based on these findings, future research should explore both digital and system-level interventions to improve AIS care. Evaluating the clinical and psychosocial effects of digital support tools, such as apps for motivation, pain management, and communication, should be complemented by studies on structural health care improvements, such as early intervention strategies, standardization of post-operative care, and expansion of multidisciplinary care models.

## Strength and limitations

The study was conducted in Norway in cooperation with the Norwegian Spine and Back Pain Association “Ryggforeningen i Norge” and scoliosis experts from different Norwegian hospitals. Therefore, the results of this study are only representative of this specific sociodemographic and geographic sample and limit generalization of our findings. The selection of the participants may have filtered out some adolescents significantly affected by AIS. Furthermore, all adolescents participants in this study were female, which may limit the applicability of the findings in contexts where gender differences are relevant. However, this sample composition is consistent with the higher prevalence and treatment rates of AIS among females. Readers should consider the transferability of findings in light of the described study population.

By incorporating multiple stakeholder groups – adolescents with AIS, caregivers, health care personnel, and IT experts – the study offers a rich, triangulated understanding of AIS‑related needs and priorities (a key strength), while we acknowledge that integrating these diverse perspectives may modestly obscure nuances unique to each group. While the inclusion of multiple stakeholder groups enriches the depth and diversity of perspectives, it also introduces variability in experiences and expectations that may limit the comparability of responses across groups.

Additionaly, the digital solution was introduced as a conceptual framework, meaning participants' feedback was based on theoretical understanding rather than extended practical use. In the absence of feasibility studies and real-world implementation, assessing the app's actual effectiveness and its capacity to address identified needs remains challenging. One limitation of this study is its qualitative design, which restricts the generalizability of the findings.

Finally, another limitation of this study is the combined analysis of adolescents, caregiver, and expert perspectives, which may have masked important differences in their experiences and priorities. Adolescents and caregivers emphasized personal and emotional aspects of AIS care, whereas health care personnel and IT experts focused on systemic and technological solutions. Additionally, the predominance of IT experts may have skewed the emphasis toward digital interventions. Future studies should consider separate analyses to better capture the distinct needs of each group.

## Conclusion

This study focuses on the management of AIS in Norway. Data obtained via focus group interviews by adolescents, caregivers, and health care personnel captured their experiences with receiving or delivering care. Adolescents and caregivers shared their frustrations and difficulties that they encountered during their journeys, such as limited access to information, poor, age-appropriate communication with health care personnel, pain management, social isolation, or lack of psychological support or motivation. It highlighted the areas in which the current AIS health care service in Norway requires improvement. These issues were openly discussed in dialogue with health care personnel and IT experts, fostering a shared understanding of existing gaps and potential solutions.

Clinical practice in Norway has changed in recent years. General guidelines have been adopted and are now being followed. Health care personnel are working to spread awareness and increase knowledge about AIS to support early detection. Adolescents are offered group therapy, and care is becoming more holistic, with psychologists included as part of a multidisciplinary team. Clinical implications now include recommendations beyond digital tools, such as enhancing early detection, broadening multidisciplinary teams to include occupational therapists, improving post-operative follow-up, including pain management, adopt comprehensive support strategies, and standardizing access to care across regions.

Our findings are applicable in clinical practice. The current health care service can be further improved using patient-centered digital technology. The possibility of implementing a digital technology in AIS treatment is expected to provide high-quality and accurate information to the adolescents at different stages of their condition, which would help adolescents to be more prepared to receive further instructions and make more informed decisions. The digital solution could include treatment tips and shared experiences from other adolescents with AIS, enabling peer connection and emotional support.

Finally, future studies could be focused on developing such a digital solution and examining its effects in clinical practice. Together, these findings suggest that meaningful improvements in AIS care will require both digital innovation and targeted enhancements to health care delivery and organization.

## Supplementary Information

Supplementary Material 1.

Supplementary Material 2.

## Data Availability

No datasets were generated or analysed during the current study.

## Abbreviations

AIS: Adolescent idiopathic scoliosis
App: Smartphone application
IT: Information Technology
SSE: Scoliosis-specific exercises
