# Supplementary material for: Understanding the needs and frustrations in adolescent idiopathic scoliosis: a qualitative interview study
Source: BMC Musculoskelet Disord. 2026 Aug 1;27:925. doi: 10.1186/s12891-026-10210-7 (PMC13717966; doi:10.1186/s12891-026-10210-7)
Supplement: Supplementary file 2 [file 12891_2026_10210_MOESM2_ESM.docx]

**Appendix 2**

**Focus group interview guide for participants**

1. **Need & Pain Points in Scoliosis Journey workshop (participants: experts, researchers, companies, user representatives from the Norwegian Spine and Back Pain Association “Rygforenningen I Norge”)**

**Patients’ journey and clinical practice**

- Can you share examples of the patient journey for the three different types of patients?
- What happens in the different stages of the patient’s journey?
- What is the current clinical practice for scoliosis treatment and what is the best clinical practice?
- What are the improvements needs for today's clinical practice from a patient perspective?

**Treatment planning and communication among experts**

- How is the treatment planned for the different patient types? What types of experts are involved in different phases (their roles)?
- How does communication occur between the experts in different phases?
- How are decisions made in different phases, what data is used for decision-making for these three different types of patients, and why?

**Patient and parental guidance**

- What kind of treatment guidelines/guidance does the patient receive in different phases?
- How are treatment guidelines and scoliosis braces given and communicated to the patients and their parents?
- What are the current challenges with the patient guidelines/guidance from the perspective of different types of patients and clinical experts?
- What are today’s challenges in communication?

**Technological solutions**

- What technology and other solutions are used nowadays, and how is the treatment provided in practice?
- What kind of technological possibilities do the experts meet in the different phases and at different patient groups?

**Challenges and needs**

- What challenges and needs do different types of patients face in different phases of the patient journey? Why?

2. **Future Solutions workshop (participants: experts, researchers, companies, user representatives from the Norwegian Spine and Back Pain Association “Rygforenningen I Norge”)**

**Use of technology in the treatment process/treatment journey**

- What technology and solution components can be used to support different patient groups and at different phases of their journey? Why?

**Motivation**

- What technology and solution components and content will motivate different types of patients, and their parents based on the experience of expert teams? (Show some prototype generated ideas based on previous discussions, go through real-life examples)
- What type of technology use will improve the experience of the different types of patients? How?
- What types of patients will benefit most from the use of technology, and why?

**Technological touch points, content options, changes, and optimization**

- What are technological touchpoints and content?
- How would the use of technology change the treatment practice?
- What can be optimized due to the use of technology?

**Use of data**

- What data can be used in decision-making?
- How can the data be used in decision-making processes in different phases?

**Communication**

- What kind of benefits can technology bring to communication between experts?
- What advantages can technology bring to communication between patients, their parents, and experts?
- What kind of advantages and disadvantages would it bring (e.g. cost advantages, etc.)?

3. **Patients’ and parents’ workshops (participants: experts, researchers, companies, user representatives from the Norwegian Spine and Back Pain Association “Rygforenningen I Norge”, 3-5 parents/teenagers with scoliosis diagnosis)**

**Process / Treatment journey**

- You have now been through the scoliosis treatment.
- What stand out for you as major events in this process in your experience? What happened in different phases?
- Can you reflect on these events, why do they stand out to you?

**Treatment experience and motivation**

- How would you describe your experience of the various phases of the treatment?
- What do you remember as the most demanding situations or pain points in each of these phases?
- How motivated were you to do the treatment? And what motivated you to do it?

**Changes**

- What would you describe as the biggest difference in your daily routine during treatment?
- How did the hospital's requirements for scoliosis treatment affect your daily routines?
- How did you feel about this?
- What kind of motivational challenges did you face, and why? Is there any way that these could have been overcome with some changes, e.g. better scoliosis, apps, games, etc,

**Guidance from the hospital**

- How did you get information about scoliosis and treatment in different phases?
- How did you get the treatment guidance in different phases?
- Who did you communicate with about the tasks and what were the challenges in that communication?

**Potential for future digital solutions**

- Do you use apps daily? Which ones? Do you play games? Which ones? Which games do you find most engaging? How long did you play them? If the exercise was part of the game, what would the game be about?
- What do you think about these possible features: instructional videos, interactive training, messaging with the therapist, diary, calendar, and anonymous meetings with other users online? Why?
- (After the prototype demonstration) What do you think of this proposed solution? What would you change? What would you add or remove?

**Influence at home**

- How would you describe your relationship with your parents, school, friends etc? Does the scoliosis have any influence on these?
- Is there any way that these people could be better involved in the process, how to involve them to motivate you?

4. **Feedback workshop (participants: experts, researchers, companies, user representatives from the Norwegian Spine and Back Pain Association “Rygforenningen I Norge”, 3-5 parents/teenagers with scoliosis diagnosis)**

- How can the prototype help patients and parents in the different stages of scoliosis treatment?
- What works? What doesn’t work and why?
- What makes you feel motivated to use it and why?
- Do you have any ideas to improve the prototype, so it becomes better, is flexible and motivating to use in a long term?

5. **Vision workshop (participants: experts, researchers, companies, user representatives from the Norwegian Spine and Back Pain Association “Rygforenningen I Norge”)**

- What possibilities are there for scoliosis solutions in the future?
- What is the expected added value it provides?
- How does this differ from existing solutions/possibilities?
- What are the funding opportunities for the main project?
- How can be this scaled up and how to achieve it in practice?
